# *BINoculars*: data reduction and analysis software for two-dimensional detectors in surface X-ray diffraction

**DOI:** 10.1107/S1600576715009607

**Published:** 2015-06-27

**Authors:** Sander Roobol, Willem Onderwaater, Jakub Drnec, Roberto Felici, Joost Frenken

**Affiliations:** a Huygens-Kamerlingh Onnes Laboratory, Leiden University, PO Box 9504, 2300 RA Leiden, The Netherlands; b ESRF, 71 avenue des Martyrs, 38000 Grenoble, France

**Keywords:** data analysis, data reduction, reciprocal-space mapping, X-ray diffraction

## Abstract

*BINoculars* is a tool for data reduction and analysis of large sets of surface diffraction data that have been acquired with a two-dimensional X-ray detector. The intensity of each pixel of a two-dimensional detector is projected onto a three-dimensional grid in reciprocal-lattice coordinates using a binning algorithm. This allows for fast acquisition and processing of high-resolution data sets and results in a significant reduction of the size of the data set. The subsequent analysis then proceeds in reciprocal space. It has evolved from the specific needs of the ID03 beamline at the ESRF, but it has a modular design and can be easily adjusted and extended to work with data from other beamlines or from other measurement techniques. This paper covers the design and the underlying methods employed in this software package and explains how *BINoculars* can be used to improve the workflow of surface X-ray diffraction measurements and analysis.

## Abstract 2

*BINoculars* is a tool for data reduction and analysis of large sets of surface diffraction data that have been acquired with a two-dimensional X-ray detector.

## 1. Introduction   

Over the past decade there have been several developments that have radically changed data acquisition in X-ray diffraction experiments. The primary development is that nearly all point detectors have been replaced by two-dimensional detectors, such as the MAXIPIX detector (Ponchut *et al.*, 2011 ▸), that collect spatially resolved information from a region in reciprocal space in a single shot. Secondly, by synchronizing the data acquisition with the actuation of the diffractometer motors, it is now possible to perform continuous scans during diffractometer movements. Even though this was demonstrated 50 years ago (Arndt & Phillips, 1961 ▸), it has only recently become routine practice (Shayduk & Braun, 2008 ▸). Thirdly, the high photon flux at third-generation synchrotrons (Winick, 1998 ▸) allows integration times of the order of tens of milliseconds rather than seconds, thus enabling time-dependent observations of dynamic processes rather than the slow acquisition of static information.

The result of these developments is that the data acquisition rate has increased by six to seven orders of magnitude, from typically 1 point per second to millions of points per second, and so the amount of data collected during one experiment has increased dramatically. Today’s computer hardware can keep up with that increased demand, but the development of software to analyse these large data sets has been lagging behind, which has kept most users from exploiting the full potential of modern surface diffraction beamlines. *BINoculars* aims to fill this gap by taking a novel approach to data reduction in surface X-ray diffraction (SXRD) experiments.

Currently, data reduction is typically performed by integrating a region of the image from a two-dimensional detector, sometimes coupled with another integration to determine the background level. Compared to a traditional point detector, this is already a step forward because a single image contains information about the signal and the background. The sample does not need to be rocked in order to determine the background signal, as in the case of a point detector, which speeds up the acquisition. The other advantages are mostly qualitative: the large acceptance angle of the detector, in combination with its good angular resolution, is very convenient during diffractometer and sample alignment, and makes it possible to visually identify peaks by their shape (*i.e.* one can easily distinguish between a powder ring, a crystal truncation rod or a region of diffuse background). *BINoculars* improves on this, and takes full advantage of a two-dimensional detector, by treating every pixel individually.

**Figure 1:**
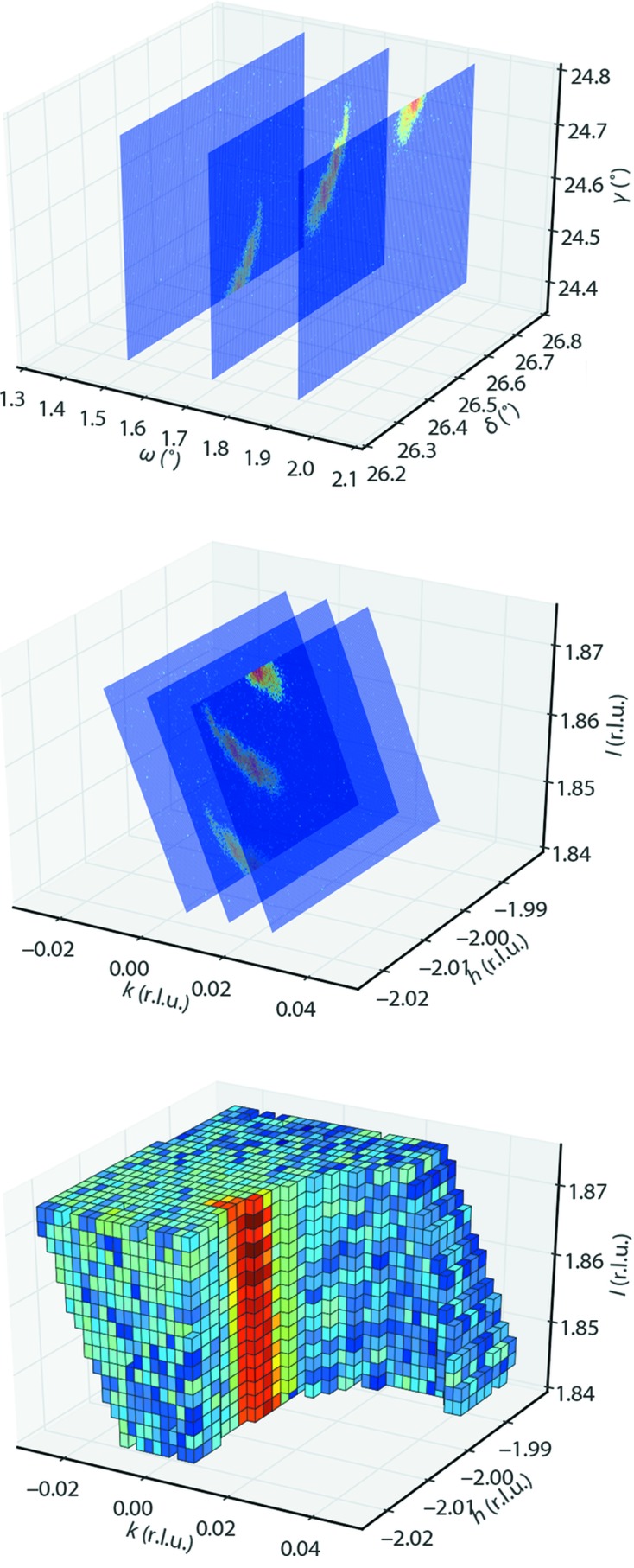
A graphical overview of the process performed by *BINoculars*. The data displayed here are a rocking scan through 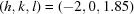, the diffraction pattern from a Pt(110) surface. Upper panel, raw data acquired by the two-dimensional detector with the corresponding angles ω, δ, γ from a six-circle diffractometer (Vlieg, 1997 ▸). Middle panel, the diffractometer angles are mapped onto reciprocal-space coordinates 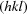. Lower panel, the averaged intensity of pixels on a regular grid of volumetric bins (voxels) is calculated. Part of the bins are removed for clarity. Note that the data in reciprocal space in the lower panel are constructed from a series of images over a larger range and with a denser sampling in ω than shown in the other panels.

*BINoculars* takes a series of images from a two-dimensional detector, calculates for each pixel the corresponding reciprocal-lattice coordinates 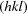, and reduces the image collection to a single data set by averaging the intensities of pixels taken at identical 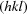 positions (within a user-specified resolution). This transformation and averaging are illustrated in Fig. 1 ▸. To allow online analysis during data acquisition, *BINoculars* can run on a high-performance cluster to process large data sets. For example, the result of a full hour of continuous scanning, adding up to a total of approximately 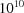 pixels, can be processed in a matter of minutes. In addition, *BINoculars* provides tools to further process the data, including visualization, curve fitting and crystal truncation rod integration. The latter can be seen as an implementation of the reciprocal-space integration method, recently described by Drnec *et al.* (2014 ▸). As a whole, *BINoculars* can be seen as an *N*-dimensional generalization of *PyFAI* (Kieffer & Karkoulis, 2013 ▸), *xrayutilities* (Kriegner *et al.*, 2013 ▸) and *FEP3D* (Gaudet *et al.*, 2013 ▸), optimized for (but not limited to) surface X-ray diffraction.

## 2. Implementation   

*BINoculars* has been written in Python, an open-source scripting language that is very suitable for scientific software, thanks to its powerful and clear syntax and the extensive support for numerical calculations *via* the *NumPy* and *SciPy* libraries (Perez *et al.*, 2011 ▸).

*BINoculars* has been designed to process large data sets and its operation is usually CPU bound. An ordinary desktop computer can easily take many hours to deal with a data set obtained in 1 h (*e.g.* 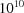 pixels with 16 bit per pixel). To allow online analysis during data acquisition at a beamline, *BINoculars* can use a computing cluster to distribute the load over multiple computers.

**Figure 2:**
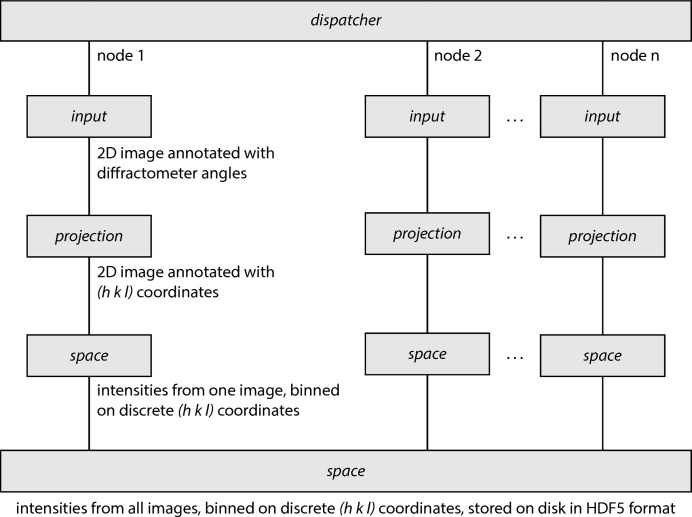
Block diagram of the process performed by *BINoculars* for a typical data set from a diffraction experiment. The *dispatcher* distributes the load over multiple nodes from a computing cluster. The *input* class gathers the experimental data and calculates the diffractometer angles for each pixel. The *projection* class converts the angles to 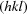 coordinates. The intensities are binned on a discrete grid by the *space* class, and finally the intermediate results from each node are combined into a single *space*.

To keep *BINoculars* modular and flexible, the workflow for processing data is separated into four modules: the *dispatcher* is in charge of the whole process and handles job parallelization and distribution, the *input* class gathers the experimental data, and the *projection* class converts the raw data into the coordinates of choice. Finally, the processed data are binned on a discrete grid and stored in a *space* class, which provides generic tools for further analysis. The specific behaviour of the *dispatcher*, *input* and *projection* modules can be changed independently. For these modules, the user can choose from several different implementations, each having a different set of features. This process is illustrated in Fig. 2 ▸.

The *input* class collects the raw two-dimensional detector images and assigns metadata to each individual pixel. These metadata are used later on by the *projection* class to make the conversion to the desired coordinate system [*e.g.* 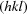 for a typical SXRD experiment]. The *input* class is specific to a certain experimental setup. As an example, for the ID03 beamline at the ESRF (Balmes *et al.*, 2009 ▸), a separate class has been written for each of the experimental hutches. In many ways the two experimental hutches are identical, for example the same numbering scheme is used for the images taken by the X-ray cameras, but one of the diffractometers operates at a constant detector–sample distance while the other does not. The *input* class takes care of all these technicalities, and writing a new *input* is the most important task when adding support for another experimental setup. In addition, the work done by the *input* class is often the most computationally intensive step in the entire process of *BINoculars*. Although it is likely that a significant performance gain can be obtained by implementing this part of the code in C rather than Python, the code is intimately related to the continuously evolving experimental practices of the beamline, and as such a high-level language is a better choice.

The next step is performed by the *projection* class, which converts the data collected by an *input* class into the appropriate coordinate system for binning. For an SXRD experiment, the *input* class will typically return a series of detector images with corresponding diffractometer angles for each pixel, and the *projection* class will convert the angles into reciprocal-space coordinates 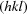 for each image. In some cases, there are several projections that are useful. For example, for the ID03 beamline it is sometimes necessary to project onto the scattering angle 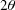 rather than 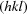 coordinates [although an alternative route would be to perform a coordinate transformation afterwards to convert the 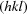 *space* into a 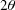 *space*].

Once the data have been gathered and projected on the desired coordinate system, the binning operation is performed by a class called *space*. This class represents an *n*-dimensional regular grid: it is a discrete subset of a vector space, where each dimension has a fixed step size. Many mathematical operations can be performed with *spaces*, including addition, subtraction, slicing, projections and coordinate transformations. To bin an image, the 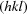 coordinates of each pixel of the image are mapped onto the nearest discrete *space* grid location. Then the pixel intensities are accumulated at every discrete grid location, using the histogram operation *bincount* from *NumPy*. In addition, the number of contributions per coordinate is stored in order to calculate the mean intensity per bin rather than the integrated intensity. This binning operation is the essential data reduction step performed by *BINoculars*: hence the name of the program.

The *dispatcher* orchestrates the entire process: it asks *input* for the sequence of images from the two-dimensional detector, delegates it to the appropriate *projection* and performs the binning operation by feeding the projection result into a *space*. Two *dispatcher* implementations are currently present: one for local processing using multiple processor cores on a single computer, and one that distributes tasks over a high-performance cluster managed using OAR (https://oar.imag.fr/). Support for other types of clusters can easily be added. When running on a computing cluster, the *dispatcher* gathers all intermediate spaces calculated by the individual nodes (for example using a shared filesystem) and they are added together to form the final resulting *space. Spaces* are stored on disk using the HDF5 file format (Folk *et al.*, 2011 ▸).

After a *space* has been created, the size of the data set has typically been reduced by a factor 10 to 100, and further analysis can usually be performed on a standard workstation. However, loading a high-resolution large-area three-dimensional data set can require several GB of memory, and for some operations it is required to have several copies in memory. If this is a problem, it is also possible to work with a subset of the data, either by selecting a smaller region or by reducing the resolution or by reducing the dimensionality.

*BINoculars* provides various tools for analysis, including mathematical operations, plotting and exporting. For ultimate flexibility it is possible to directly manipulate a *BINoculars* space from a Python script, but in many cases the standard tools are sufficient. In addition, a fitting and integration tool is available to calculate the structure factors of a crystal truncation rod to be directly inserted into the fitting program *ROD* (Vlieg, 2000 ▸). It takes as input a reciprocal mesh which it slices by a user-specified resolution and the resulting data are either fitted (typically with a two-dimensional Lorentzian) using a least-squares optimization or simply integrated. The error is estimated from equation (2), as will be described in more detail in the next section.

## 3. Binning and error handling   

*BINoculars* calculates the average intensity of multiple contributions, originating from different pixels and/or detector positions, to a single reciprocal-space bin. This operation is similar but not identical to averaging a series of repeated measurements taken by a point detector at a fixed position. This section discusses the implications of the binning operation for the background intensity and the estimation of statistical errors.

When using a point detector, the typical surface diffraction experiment is set up such that there is a unique detector position for each set of reciprocal-space coordinates 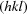. In practice, this means reducing the degrees of freedom of the diffractometer to three, *e.g.* by working with a constant surface normal and a fixed angle of incidence. When taking series of repeated observations at a certain reciprocal-space location, the systematic error in each measurement can be assumed to be constant (after the usual correction for variations in the total beam intensity), and the only variation is given by the shot noise of the incoming photons.

Using a two-dimensional detector, the spatial extent of the detector introduces two more degrees of freedom, meaning there is no longer a unique detector position for a given reciprocal-space coordinate. This is usually solved by selecting one pixel of the detector to correspond with ‘the detector position’, and ignoring the fact that the other pixels are at a slightly different position. However, *BINoculars* does take the spatial extent of the detector into account, and calculates the average intensity at each location in reciprocal space, regardless of the detector position.

**Figure 3:**
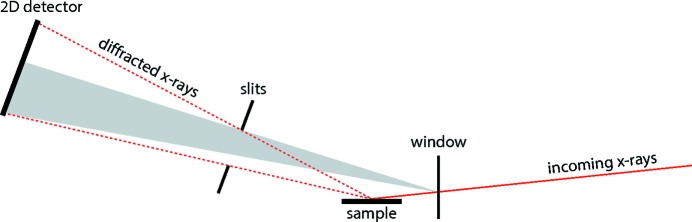
The wide opening angle of the slits, which is required to capture a region in reciprocal space with a two-dimensional detector, results in a non-uniform background across the detector (indicated in grey in the figure). This background originates from scatterers other than the sample, for example a beryllium window. This means that when taking two images at slightly different detector positions, such that there is some overlap between the two captured regions, the background intensity in the overlapping region is not constant.

This means that multiple measurements, even when spaced closely together in time, exhibit variations not only due to the statistical nature of the process but also due to a systematic error, possibly resulting from different detector positions. This error is caused by differences in background originating from scatterers other than the sample, as is illustrated in Fig. 3 ▸. Flight tubes and slits between the sample and the detector can be used to reduce this background, but they also decrease the aperture of the two-dimensional detector. This reduces the range of 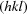 locations over which data can be collected in one acquisition. This means that a careful trade-off needs to be made between acquisition speed and background suppression.

In some cases it is possible to subtract the background 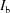, either by measuring it directly when the sample is not in the beam or by estimating it from the data set itself in regions in reciprocal space where the sample only weakly contributes to the total observed intensity. The latter approach will be explored in more detail in §4.

The remaining error reflects the counting statistics in the number of detected photons and is typically assumed to obey Poisson statistics (Robinson, 1991 ▸). For a single observation of *I* counts, the standard deviation σ is estimated using 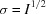. With *N* independent observations 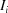 in a single bin, each with its own 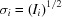, the average intensity is
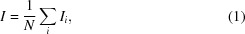
and the variance can be estimated under the assumption of normality (*N* or 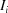 sufficiently large) using 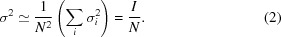
Assuming we have a separate estimate of the background intensity 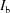 in this bin with a corresponding variance 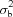, the variance 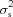 of the signal 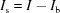 is now given by 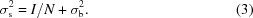
Of course, if the background was also obtained by averaging *N* measurements, 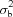 would also take the form 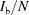.

## 4. Demonstration   

Four different examples will be discussed to show the capabilities and limitations of *BINoculars*.

**Figure 4:**
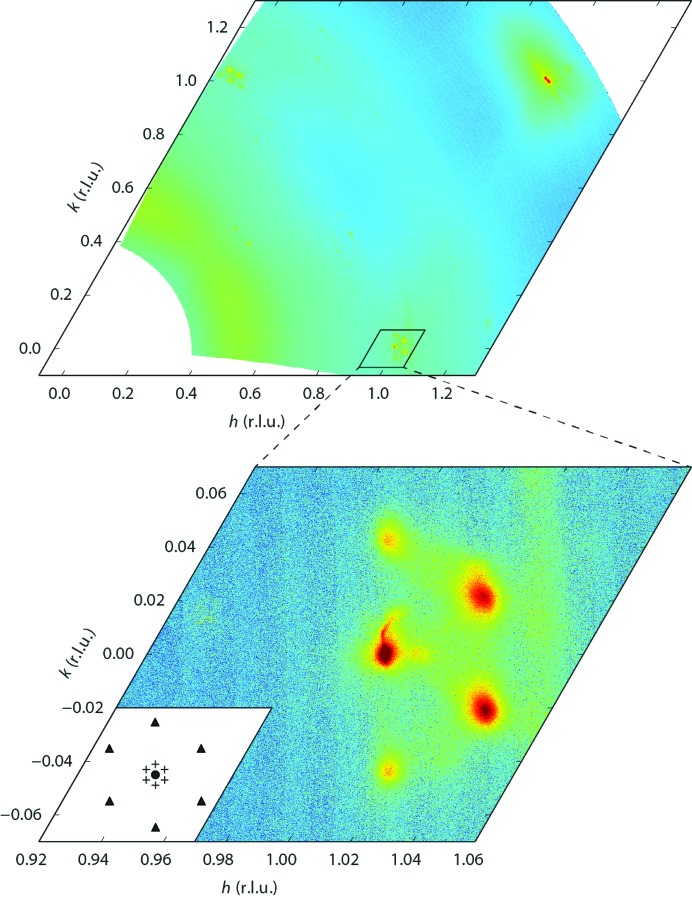
A large-area survey (upper panel) in reciprocal space of the herringbone reconstructed Au(111) surface, taken at 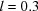. The data set has been obtained in 111 min using a series of continuous-acquisition ω scans. A zoom-in (lower panel) around the 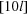 crystal truncation rod (indicated by the circle in the inset) shows the satellite peaks caused by the 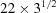 unit cell (indicated by triangles) and those originating from the longer range ordering of the zigzag domains (indicated by crosses). Loading this data set requires 1 GB of memory.

Fig. 4 ▸ shows a high-resolution (0.0002 reciprocal-lattice units or r.l.u.’s), large-area 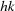 surface in reciprocal space, covering the first reciprocal unit cell of an Au(111) surface, submerged in an electrochemical cell filled with sulfate-containing electrolyte (pH 7) and kept at −800 mV *versus* Ag/AgCl reference electrode. The gold surface exhibited the so-called herringbone reconstruction (Barth *et al.*, 1990 ▸), which is a regular structure with a (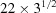) periodicity that is organized into a zigzag pattern on an even larger scale. The 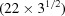 superstructure peaks originating from this reconstruction (Steadman *et al.*, 2000 ▸; Peters *et al.*, 2000 ▸) are well resolved in the scan. The differences between this diffraction pattern and the pattern reported in the literature for the same surface in ultra-high vacuum are not fully understood; however, they are likely to be due to the sample preparation procedure in the electrochemical cell. The data set was acquired in just 111 min.

**Figure 5:**
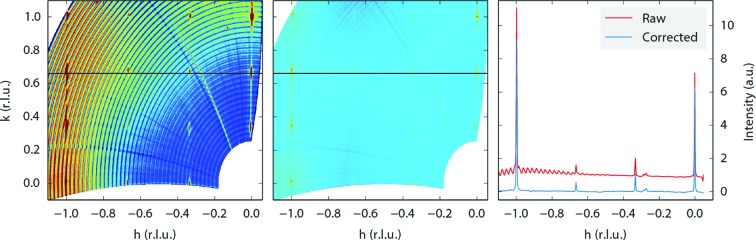
Some data sets require further processing to remove the curved background artefacts. This figure shows the 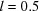 plane from a Pt(110) sample in the high-pressure flow reactor setup at ID03. The surface exhibited a 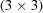 reconstruction (which has not been described before in the literature) during high-temperature high-pressure exposure to NO and H_2_. The setup had a relatively high diffuse background that could be corrected for by estimating the background level for each pixel of the detector, once per scan in ω. The left panel shows the raw data, the middle panel the data after background correction. The right panel shows the intensity profiles along the line 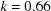 for a direct comparison between raw and corrected data. The small peaks at 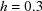 originate from scattering from the Be dome; this is also visible in the left and middle panel as a diagonal line.

Fig. 5 ▸ shows a data set that is strongly affected by background intensity. Like Fig. 4 ▸, the data set is built up from a series of ω scans, and in this case those scans are clearly visible as arcs after processing by *BINoculars*. The problem is that the background intensity depends not only on the position of a pixel but also on the position of the camera. In other words, when moving the camera by only a small amount, such that a certain feature remains in the field of view (in this case a move in γ between the consecutive ω arcs), the contribution of the background intensity to that feature can change significantly, as illustrated in Fig. 3 ▸.

This particular data set has been obtained using the high-pressure flow reactor setup at ID03 (van Rijn *et al.*, 2010 ▸), which has a beryllium dome around the sample. The dome acts as a strong X-ray scatterer only 14 mm away from the sample. It is not possible to lower the resulting background intensity using slits without dramatically reducing the aperture of the detector. However, for this sample it proved possible to estimate and subtract the background level, the result of which is shown in the middle panel of Fig. 5 ▸. For each pixel of the detector, the background was estimated as the average intensity of that pixel in all images in a single ω scan. The average is calculated by fitting a Poisson distribution, as this turned out to give better rejection of outliers (which are in fact the diffraction peaks) than a simple mean or median calculation. This process was then repeated for each ω scan, resulting in an estimate of the background intensity that was subtracted from the raw data.

The background subtraction procedure described here is not generally applicable as powder rings are treated as background. In addition, the amount of background is strongly dependent on the experimental setup, and the other data sets shown here did not need any background correction. Therefore this procedure is currently not part of *BINoculars*, although it is likely to become part of the ID03-specific *input* module in the near future.

**Figure 6:**
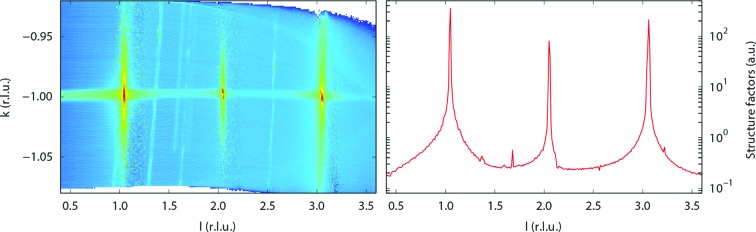
The left panel shows an *l* scan along the 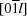 crystal truncation rod of a SrTiO_3_ (100) surface (Salluzzo *et al.*, 2013 ▸) projected on the *kl* plane after processing by *BINoculars*. A series of two-dimensional Lorentzians has been fitted to slices at constant *l* (corresponding to small *hk* surfaces), which has resulted in the structure factors shown in the right panel.

The third example, Fig. 6 ▸, shows the output of the crystal truncation rod (CTR) fitting module. The original data set is a single *l* scan along the CTR. After processing by *BINoculars*, during which the *input* module also takes care of the polarization correction factor necessary to obtain the structure factors (Vlieg, 1997 ▸), the three-dimensional rod can be visualized in reciprocal space. The data set is then segmented into small intervals along *l*. Each section is analysed separately by a numerical integration algorithm to calculate the structure factors as a function of *l*. This method has recently been described by Drnec *et al.* (2014 ▸).

At lower *l* the detector is more perpendicular to the surface, and for some samples it might be useful to augment the *l* scan with rocking scans at low *l*. *BINoculars* can easily deal with such a hybrid data set, since it starts by processing the data into the three-dimensional rod, after which the integration procedure (taking place in reciprocal space) is performed completely independently of the original character of the raw data.

**Figure 7:**
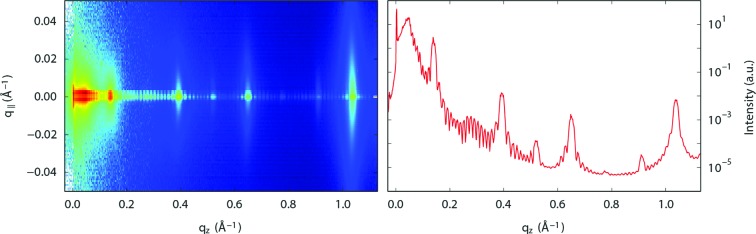
Reflectivity scan of (PbSe)_4+δ_(TiSe_2_)_4_, a telluride misfit layer compound (Moore *et al.*, 2014 ▸). This sample has a multilayer structure with well defined out-of-plane stacking but rotational disorder between layers. This gives rise to oscillations in the reflectivity curve, combined with broad in-plane scattering. The left panel shows the projection of the images taken at different incident angles onto 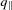 and 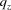. The right panel shows the specular rod obtained by integration along 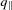 from −0.005 to 0.005 Å^−1^.

The fourth example demonstrates that *BINoculars* can be used with other coordinate systems than 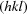. Fig. 7 ▸ shows the reflected intensity from a (PbSe)_4+δ_(TiSe_2_)_4_ sample. It is constructed from images taken at different incidence angles that are projected onto 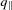 and 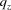, the in-plane and out-of-plane momentum transfer. This two-dimensional projection allows detailed analysis of the in-plane X-ray scattering that cannot easily be obtained by conventional methods. In addition, the integration to obtain the specular rod (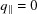) can be easily performed.

## 5. Conclusion   

*BINoculars* unlocks the full power of two-dimensional detectors and helps visualize surface diffraction patterns to an extent that was previously only achievable with low-energy electron diffraction in ultra-high vacuum conditions, or by increasing the X-ray energy and sacrificing *k*-space resolution and dynamic range (Gustafson *et al.*, 2014 ▸), or by taking an impractical amount of time. In addition, *BINoculars* simplifies structure determination by providing a quick, easy and accurate method to integrate crystal truncation rods.

*BINoculars* is open source under the terms of the GNU General Public Licence (http://www.gnu.org/licenses/gpl.html) and is available online (http://binoculars.uithetblauw.nl/; https://github.com/id03/binoculars).
